# Genomics applied to livestock and aquaculture breeding

**DOI:** 10.1111/eva.13378

**Published:** 2022-04-18

**Authors:** José M. Yáñez, Peng Xu, Roberto Carvalheiro, Ben Hayes

**Affiliations:** ^1^ Facultad de Ciencias Veterinarias y Pecuarias Universidad de Chile Santiago Chile; ^2^ 12466 Fujian Key Laboratory of Genetics and Breeding of Marine Organisms College of Ocean and Earth Sciences Xiamen University Xiamen China; ^3^ Departamento de Zootecnia Faculdade de Ciências Agrárias e Veterinárias UNESP – Univ Estadual Paulista Jaboticabal Brazil; ^4^ CSIRO Agriculture & Food Hobart Tas Australia; ^5^ 1974 Centre for Animal Science Queensland Alliance for Agriculture and Food Innovation The University of Queensland Brisbane Qld Australia

**Keywords:** aquaculture, genomics, livestock, selective breeding

## Abstract

The increasing global demand for food, due to the continuous growth of human population, requires improvements in the efficiency and sustainability of animal production systems. In addition, several challenges facing farming of aquatic and terrestrial organisms need to be overcome to ensure food security in the upcoming decades, e.g. adaptation to climate change, reduced availability of conventional animal feed ingredients, emerging infectious and parasitic diseases, among others. Genomic technologies such as massive parallel sequencing, high‐throughput genotyping, genome selection and gene editing, combined with highly efficient computational methods can accelerate the rate of genetic progress in animal breeding. Thus, such technologies can help us meet the needs for protein sources for human consumption in the upcoming years. This Special Issue aims at presenting current advancements in the field of genomic tools applied to aquatic and terrestrial farmed animal populations.

## 1 INTRODUCTION

The increasing global demand for food, due to the continuous growth of human population, requires improvements in the efficiency and sustainability of animal production systems. In addition, several challenges facing farming of aquatic and terrestrial organisms need to be overcome to ensure food security in the upcoming decades, e.g., adaptation to climate change, reduced availability of conventional animal feed ingredients, emerging infectious and parasitic diseases, among others. Genomic technologies such as massive parallel sequencing, high‐throughput genotyping, genome selection and gene editing, combined with highly efficient computational methods can accelerate the rate of genetic progress in animal breeding. Thus, such technologies can help us meet the needs for protein sources for human consumption in the upcoming years. This Special Issue aims at presenting current advancements in the field of genomic tools applied to aquatic and terrestrial farmed animal populations. From a general stand point, this Special Issue provides a better understanding on the utilization of genetic variability to improve aquaculture and livestock populations through the use of genomics. The contributions presented here demonstrate the utility of genomics in studying a variety of aspects of quantitative traits and selection applied to aquatic and terrestrial farmed species, including: (i) assessing genetic diversity of populations by using high‐resolution genomic information, (ii) identifying the genetic architecture of important quantitative traits, (iii) leveraging genome‐wide information for genetic evaluations, (iv) understanding genomic variation shaped by domestication and selection, (v) integration of different sources of information (e.g. non‐coding DNA sequences) to uncover mechanisms underlying complex traits, as well as, (vi) development and application of novel statistical methods for the analysis of genetic information.

## 2 ANIMAL GENETIC IMPROVEMENT

The application of genetic improvement has for decades been one of the most efficient tools to increase the biological performance and sustainability of animal production systems in the majority of terrestrial species (Goddard & Hayes, 2009). However, aquaculture species are, in general, behind terrestrial farm animal industries regarding selective breeding practices. For instance, it has been estimated that less than 10% of aquatic animal farming is based on genetically improved populations (Gjedrem, 2012). Nevertheless, response to selection documented for aquaculture species are in general considerably higher than that of livestock and there is growing interest to increase positive economic impact via selective breeding programs in aquatic farmed animals (Gjedrem et al., 2012). The status of selective breeding programs and technology level used in terrestrial species is very advanced and more or less similar among the most industrialized production species, including pigs (*Sus scrofa domestica*), chicken (*Gallus gallus domesticus*) and cattle (*Bos* spp.; Georges et al., 2019). However, aquaculture production is based on a wide range of technological input and genetic development levels, from the use of non‐domesticated seed stocks from the wild through the implementation of breeding schemes leveraging genomic information (e.g., genomic selection; Houston et al., 2020; Yáñez et al., 2015).

## 3 GENOMIC TECHNOLOGIES

The development of genomic tools such as reference genome sequences, massive parallel re‐sequencing approaches and dense single nucleotide polymorphisms (SNP) genotyping panels have been crucial to both improve basic knowledge on the genomic control of complex traits and increase the rate of genetic progress in livestock and aquaculture species. In fact, genomic technologies are currently being routinely used in selective breeding to support livestock production, including species such as pigs, chicken and cattle (Georges et al., 2019), as well as in industrialized aquaculture species, including Atlantic salmon (*Salmo salar*), coho salmon (*Oncorhynchus kisutch*), rainbow trout (*Oncorhynchus mykiss*; Lhorente et al., 2019) and tilapia (*Oreochromis* spp.; Yáñez et al., 2020). Thus, genomic advancements provide powerful tools for the scientific community and food industry to meet global demands of animal protein supplies for human consumption during the next years. The power of genomics is particularly important in livestock and aquaculture. The latter can be considered a young and rapidly expanding activity, with no more than 50 years of industrialized history, and an extremely unbalanced scientific and technological development in selective breeding practices across species.

## 4 DOMESTICATION AND ARTIFICIAL SELECTION

Domestication of terrestrial animals has been a long‐term ancient process. For the most important livestock species, domestication begun ~10,000 year ago, most likely starting as a commensal interaction between wild ancestors of current domestic animals and humans, followed by an increasingly intent to keep control of production, intensively breed and at last commercialize the use of animals by humans (Zeder, 2012). During this long‐term process, several domestic breeds have been established for different animal species across the world. Some breeds have been intensively selected for specific purposes resulting in highly divergent stocks within the same species. For instance, some chicken breeds are highly specialized for their use in intensive meat (broilers) or egg (layers) production. Similarly, some cattle breeds have been highly selected for either milk (dairy) or meat (beef) production. Genomic technologies can be used to investigate different demographic events that occurred across the domestication history of animal populations.

Cattle can be currently considered the economically most relevant domestic animal in the world. A comprehensive mitogenome assessment of the bovine species, including 114 breeds, suggests that the increase in the effective female population size of different bovine haplotypes overlaps with important environmental and anthropic events, such as climate changes at the end of ice age, the beginning of domestication, and migration of farmer tribes in Africa. Moreover, the study supports the presence of rare aurochs (*Bos primigenius*) female‐mediated adaptive introgression in domestic cattle during the dispersal of the species in Europe (Cubric‐Curik et al., 2021).

A comprehensive genome‐wide survey of worldwide chicken breeds provided deep insights into the admixture events which have shaped the genome of modern chicken populations during the domestication process, indicating that animals used for meat production in Asia, Europe and the US are highly represented by genetic contributions from heavy Asian breeds as well as confirming the molecular basis of plumage pigmentation (Guo et al., 2021). Another bird species which has been bred for different purposes, including ornamental, production, competition, and experimental uses, is the domestic pigeon (*Columbia livia*). By using whole‐genome sequences of animals from seven breeds it has been revealed that the genetic diversity and inbreeding levels of domestic pigeons are consistent with human‐driven selection, indicating that genomic regions under selection in commercial populations for production harbor genes associated to body size, reproduction, and feather color; while in ornamental populations genes are mostly related to skeletal‐muscle features, potentially affecting appearance traits (Hou et al., 2021). These studies provide further insights into the impact of domestication and artificial selection in shaping the genome of terrestrial domestic animals, which represent valuable knowledge for within‐species genetic diversity conservation and selective breeding.

History of domestication and artificial selection in aquaculture species is much more recent, and in some cases very incipient (López et al., 2015). Selection signatures, generated during the early domestication and recent selective breeding, have been assessed at a genome‐wide scale in important farmed fish species, including Atlantic salmon (Gutierrez et al., 2016; López, Benestan, et al., 2019; López, Linderoth, et al., 2019; Naval‐Sanchez et al., 2020), rainbow trout (Cádiz et al., 2021), coho salmon (López et al., 2021), Nile tilapia (*Oreochromis niloticus*; Cádiz et al., 2020; Hong Xia et al., 2015), channel catfish (Sun et al., 2014), common carp (*Cyprinus carpio*; Xu et al., 2019), turbot (*Scophthalmus maximus*; Aramburu et al., 2020), Australasian snapper (*Chrysophrys auratus*; Baesjou & Wellenreuther, 2021), gilthead seabream (*Sparus aurata*; Gkagkavouzis et al., 2021) tambaqui (*Colossoma macropomum*; Agudelo et al., 2022), as well as in shellfish farmed populations, including bay scallop (*Argopecten irradians*; Wang et al., 2021), Yesso scallop (*Mizuhopecten yessoensis*; Lv et al., 2022), European flat oyster (*Ostrea edulis*; Vera et al., 2019) and Pacific oyster (*Crassostrea gigas*; Hu et al., 2021; Jiao et al., 2021). These studies have used dense genome‐wide genotypes to identify genomic regions harboring genes affecting important phenotypes in farmed fish and shellfish. In general, studies on genomic footprints of domestication and artificial selection in aquaculture species have identified loci, which are putatively associated to adaptation to different biotic and abiotic conditions imposed by farming and management practices, as well as traits improved by selective breeding. Genes enriched within the loci underlying selection aquaculture populations are generally associated with growth, immune response, behavior and reproduction, indicating that these processes are important during the adaptation to captivity and also favored by recent human‐driven selection.

## 5 MODERN SELECTIVE BREEDING

Over the past decade, genomic information has been introduced in selective breeding programs for the most important terrestrial livestock species, doubling the rate of genetic progress in some cases (Georges et al., 2019). Similarly, there has been a rapid commercial implementation of genomic approaches to both investigate the genetic basis and accelerate the genetic progress of economically important traits in major industrial aquaculture species including Atlantic salmon (Verbyla et al., 2021), coho salmon, rainbow trout (Lhorente et al., 2019), tilapia (Yáñez et al., 2020), and large yellow croaker (*Larimichthys crocea*) (Zhao et al., 2021). However, and although routine implementation of genomic selection is not yet common in most of the aquaculture species, recent scientific and technological efforts aiming at diversifying aquaculture are accompanied by advancements in genomics to assist genetic assessment of farmed stocks and selective breeding. For instance, a strong candidate species for diversifying cold‐water aquaculture is Arctic charr (*Salvelinus alpinus*). A genomic characterization of the genetic diversity and evaluation of economically important traits has been recently performed in Nordic farmed populations of Arctic charr by using a genotyping‐by‐sequencing approach (Palaiokostas et al., 2021). A similar approach has been also used to investigate genetic diversity and genetic variation for growth‐related traits in a captive population of silver trevally (*Pseudocaranx georgianus*), which is considered a promising emerging for New Zealand aquaculture (Valenza‐Troubat et al., 2021). Similarly, the first application of genomic selection and genome‐wide association studies for growth traits in rock bream (*Oplegnathus fasciatus*), a valued species for marine aquaculture in Asia, has been recently reported (Gong et al., 2021). Incorporation of genomic information can also be a useful approach to assess the levels of inbreeding, their effect on economically important traits and even the genomic regions affecting changes in performance of breeding populations, as it has been recently demonstrated for female size and reproduction traits in rainbow trout (Paul et al., 2021).

To leverage the advantages offered by the incorporation of genomic information into breeding programs, generally genotyping several thousands of animals on each generation is required. The extra cost of generating genomic data can be not affordable for small‐scale livestock and aquaculture producers. Therefore, the cost of genotyping is considered a strong limitation for the routine implementation of genomic selection in low‐input smallholder animal production systems (Calus et al., 2014; Carvalheiro et al., 2014; Cleveland & Hickey, 2013; Yoshida et al., 2019). Thus, while genomic selection has been fully deployed in highly developed and industrialized animal production sectors, genomic approaches are yet to be implemented into breeding programs for several small‐scale production systems. Cheaper low‐density SNP panels (i.e. hundreds of markers), combined with genotype imputation strategies can potentially represent a cost‐effective approach to incorporate genomic information compared with using more expensive higher‐density SNP panels (i.e., thousands of markers). In fact, imputation from low‐ to high‐density genotypes have been demonstrated to provide similar accuracy levels of genomic selection to those obtained with the use of high‐density SNP panels only in livestock (Berry & Kearney, 2011; Cleveland & Hickey, 2013; VanRaden, 2020) and aquaculture species (Song and Hu, 2021; Yoshida et al., 2018), thus, decreasing the costs of genotyping and making genomic selection more affordable.

The use of ultra‐dense genomic information available from re‐sequencing data has been extensively evaluated in livestock to increase resolution of GWAS and improve accuracy of genomic selection (Ni et al., 2017; Raymond et al., 2018; Song et al., 2019). Despite that in aquaculture species the use of sequence data has not been widely applied, there is increasing interest to incorporate high‐resolution information in genomic applications to boost genetic improvement. For instance, a recent work showed an improvement in the accuracy of genomic selection for tolerance to chronic thermal stress in rainbow trout by using genetic variants from imputed re‐sequencing data and prioritized from GWAS (Yoshida & Yáñez, 2021). It is expected that in the near future more studies evaluating the potential of whole‐genome sequence data for genomic applications are available for farmed fish and shellfish.

## 6 AREAS FOR INNOVATION

Now that genomic selection has been increasingly and extensively adopted in aquaculture and livestock, the understanding of the current limitations is better acknowledged and there is renewed interest in the next steps for further innovation by the industry and scientific community. For instance, the identification, validation, and incorporation of causative variants into genomic selection models is one of the topics attracting great attention in animal breeders, because this source of information is expected to increase accuracy of selection and the rate of genetic progress (Pérez‐Enciso et al., 2015). Gene editing (e.g., CRISPR–Cas9) technology represents a powerful tool to investigate and exploit functional variants associated with complex traits for livestock and aquaculture breeding (Ruan et al., 2017).

Today, we also know that the molecular control of the expression of quantitative traits might be highly complex. A better understanding of the mechanisms involved in the gene regulation of complex traits comes from transcriptomic studies, which evaluate not only gene expression but also the role of non‐coding transcripts in trait variation. For example, it has been recently shown that differentially expressed long non‐coding RNA might be affecting meat quality traits in cattle, suggesting that specific transcripts have a role in tenderness and marbling (Muniz et al., 2022). Additional improvements required to make further advancements in selective breeding are not only related with the integration of novel sources of “omics” information but also related with the development of improved statistical approaches which may account, for example, for indirect genetic effects on the heritable variation of economically important traits (e.g., social effects), which might affect response to selection (Marjanovic et al., 2022).

## 7 CONCLUSION

The livestock and aquaculture industries have been visionary and innovative in the application of genomic technologies to improve production through selective breeding. The articles available in this special issue represent excellent examples of the current status in the application of genomics in animal breeding and also potential developments for further improvements. The utilization of genomic approaches to both evaluate and exploit heritable variation for key traits through genomic‐assisted breeding programs is paramount to the continued development and stability of worldwide animal production.
